# Synchronous Presentation of Cavernous Sinus Metastasis and Cervical Cancer: A Case Report and Review of Literature

**DOI:** 10.14740/wjon854w

**Published:** 2014-12-03

**Authors:** Sharang Tenjarla, Sheema Chawla, Eugene P. Toy

**Affiliations:** a Department of Radiation Oncology, Rochester General Hospital, 1425 Portland Ave., Rochester, NY 14621, USA; b Department of Obstetrics and Gynecology, Rochester General Hospital, 1425 Portland Ave., Rochester, NY 14621, USA

**Keywords:** Cavernous sinus metastasis, Brain metastasis, Cervical cancer, Sixth nerve palsy

## Abstract

Uterine cervical cancers customarily spread by local extension to the adjacent viscera or through lymphatic embolization to the retroperitoneal lymph nodes. Distant hematogenous spread particularly to the brain is an uncommon and late event which heralds a poor prognosis. We report a case of uterine squamous cell carcinoma of the cervix with unusual simultaneous presentation of cavernous sinus metastasis and multiple brain metastases while reviewing the current literature on this clinical entity.

## Introduction

Metastatic brain tumors are the most common intracranial neoplasms in adults accounting for 40-50% of brain tumors. Advances in neuroimaging including computed tomography (CT) scans and magnetic resonance imaging (MRI) have led to early and more frequent detection resulting in improved survival of patients. The majority of brain metastases originate from one of the three primary malignancies: lung cancer (40-50%), breast cancer (15-25%) and melanoma (5-20%) [1]. Other malignancies with metastatic potential to the brain include bladder cancer, kidney cancer, leukemia and lymphoma. Brain metastasis from gynecological cancer is a rare phenomenon with the exception of choriocarcinoma, where a study showed that the autopsy incidence of brain metastasis was 66.7% in 24 patients of these cases [2]. In contrast, brain metastasis from cervical cancer is a rare event, occurring in 0.5-1.2% of patients with cervical cancer as shown by various studies [3, 4]. They generally carry a poor prognosis with limited survival.

The present case report describes an unusual presentation of advanced squamous cell carcinoma of the cervix with cavernous sinus metastasis and concomitant multiple brain metastases while reviewing the current literature and management of this rare diagnosis.

## Case Report

A 45-year-old Caucasian lady having a past medical history of obesity, type II diabetes and hypertension presented with bleeding per vagina, hematuria, low urine output, intermittent headache, ataxia, diplopia and blurring of vision. She also experienced a weight loss of 60 pounds over 1 year, shortness of breath on exertion and a dry cough. The patient was treated with multiple antibiotics for a suspected urinary tract infection (UTI). Following an abnormal Pap smear, her vaginal examination revealed a hard mass in the cervix extending to the left vaginal wall, left pelvic wall and inferiorly to the lower vagina and introitus, suspicious of a cervical primary. Cervical biopsy revealed multiple nests of markedly atypical squamous cells and an ulcer bed with numerous deep nests of atypical squamous cells amidst extensive necrosis consistent with invasive poorly differentiated squamous cell carcinoma. Immunohistochemical study showed the tumor cells to be P16-positive and the nests of the invasive tumor were highlighted by pancytokeratin immunostain.

Physical examination revealed a pale appearing lady with an ECOG performance status of 2. Neurological evaluation revealed complete restriction of abduction in the right eye indicating sixth cranial nerve palsy and gait ataxia. In addition, there was a 2 cm × 1.5 cm firm, palpable, ovoid mass on the right side of her scalp, which on biopsy showed metastatic poorly differentiated squamous cell carcinoma consistent with origin from the uterine cervix.

Laboratory analysis showed leukocytosis, a low hematocrit and acute renal failure with creatinine of 3.4 and UTI. She was given intravenous fluids, blood transfusion and antibiotics.

**Figure 1:**
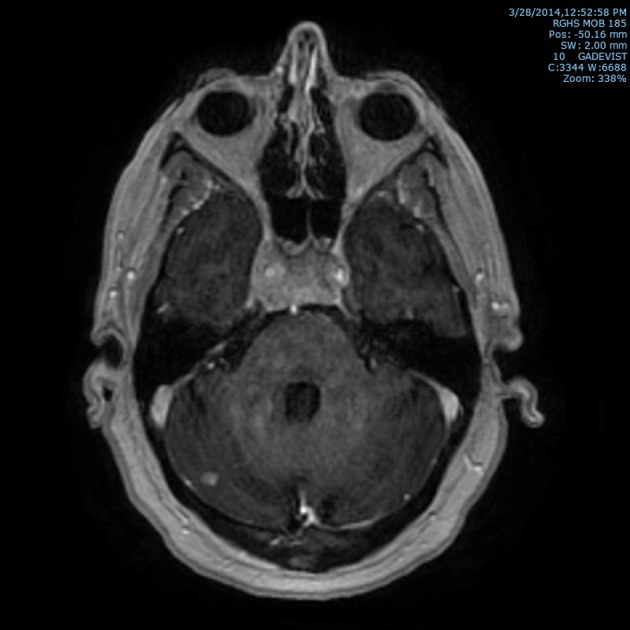
T1 post-contrast axial MRI image of brain showing marrow signal abnormality within the clivus with an expansile mass with soft tissue component in the cavernous sinus and right cerebellar metastasis.

**Figure 2:**
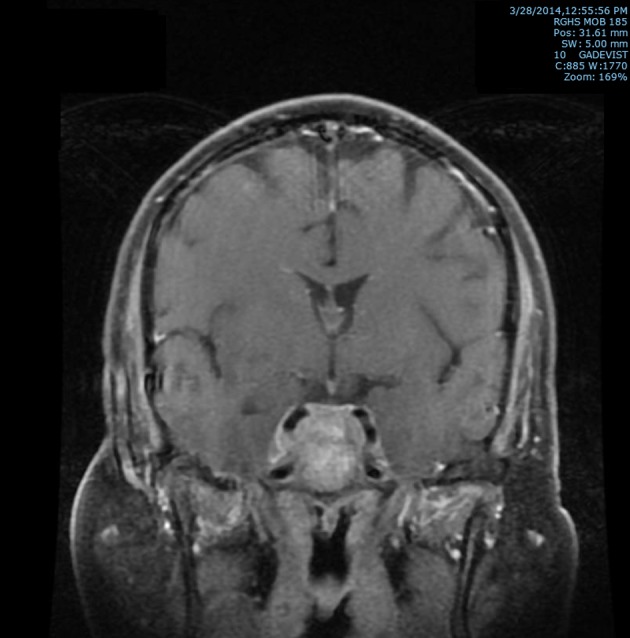
T2 post-contrast coronal MRI image of brain showing extension superiorly into the sella and posteriorly into the prepontine cistern with soft tissue prominence of right cavernous sinus with associated encasement of the right internal carotid artery.

Subsequently, an MRI of the brain showed multiple intraaxial enhancing lesions involving the frontal lobes, cerebellar hemispheres, right occipital lobe in addition to an enhancing, expansile mass in the clivus with soft tissue component in the right cavernous sinus extending anteriorly into the sphenoid sinus (Fig. 1), superiorly into the sella and posteriorly into the pre-pontine cistern. There was encasement of the right internal carotid artery and presence of moderate associated vasogenic edema (Fig. 2). All of these findings were most suggestive of intracranial metastatic disease. Due to the presentation of multiple metastatic sites in the brain, it was not felt necessary to biopsy the cavernous sinus lesion.

CT of abdomen and pelvis showed mild to moderate right hydronephrosis, a suspicious right hepatic lobe mass, and an enlarged uterus. Chest CT revealed innumerable pulmonary nodules with enlarged mediastinal and bilateral hilar lymph nodes, metastatic disease being the leading consideration. Presumptive diagnosis of advanced squamous cell carcinoma of cervix with brain, skin, lung and possible liver metastases was made. Cystoscopy and stent placement was attempted for right hydroureteronephrosis but apart from irrigation of blood clots a stent could not be passed due to complete occlusion of the right ureteric orifice.

For her brain metastases, the patient was initially given IV followed by oral dexamethasone. She was planned for palliative radiation to the brain because of multiple, unresectable brain metastases. She underwent CT-based simulation of her brain to delineate target volumes and critical structures. Her whole brain was treated using parallel opposed fields using 6 MV photons to a dose of 30 Gy at 2.5 Gy per fraction in 12 fractions in 2.5 weeks. Thereafter, the plan was to perform a boost to a large metastatic lesion in the right cavernous sinus region, which was causing compression of her sixth nerve to a dose of 5 Gy at 2.5 Gy per fraction; however, the patient could not complete this treatment because of rapid deterioration in her symptoms, septicemia and mental status changes. Likewise, local radiation or chemotherapy for her local and systemic spread could not be performed. She was sent for comfort care to hospice. She passed away exactly a month after palliative whole brain radiation.

## Discussion

Carcinoma of cervix preferentially spreads by direct extension to the adjacent organs and sequentially through lymphatics to the pelvic and subsequent para-aortic lymph nodes. Hematogenous metastasis is a less common route of spread [4-8]. The common sites of distant metastasis are lung, supraclavicular lymph nodes, liver, and bones [6]. Less common sites reported are the skin, thyroid, pleura, heart, spleen, and introitus [7]. The brain is a rare site of metastasis. Henriksen [9] first reported brain metastases from cervical carcinoma in an autopsy study in 1949. Few reports have been published since then on this clinical entity. The incidence of brain metastases in this setting has been reported to be about 0.5-1.2% in various clinical studies of patients with cervical cancer [3, 4]. Over the years, a rise in the prevalence of brain metastasis from cervical cancer has been observed. This increase may be attributed to improvement in imaging and survival as a result of more effective treatment [10].

Previous reports have shown that advanced disease, bulky tumor, endometrial extension, and lymph node metastasis are associated with an increased incidence of brain metastasis in cervical cancer [4, 11, 12]. Our case presented with locally advanced, bulky disease with metastases to lung, skin and possibly liver. Brain metastases are more frequently seen with poorly differentiated cervical tumors [13] and neuroendocrine carcinoma [14]. In our case, both the primary and the metastatic skin tumor had a poorly differentiated histology.

The presence of tumor emboli in the cerebral circulation does not necessarily culminate in the development of brain metastasis. The development of brain metastases depends on the host immune response, tissue neovascularization, the number of tumor emboli, and characteristics of the tumor [15]. The vertebral venous system has been suggested to be the main route of brain metastases by Chura and colleagues [16]. The vast majority of cases of brain metastases from cervical cancer are located in the supratentorial region of the brain, a phenomenon that may be related to the vascularity and the spatial characteristics of this region [4, 5, 14]. Similarly, in our case most of the metastases were located in the supratentorial region. Signs and symptoms depend on the location of brain metastases and the surrounding vasogenic edema and are sudden in onset, but headache and hemiparesis are most commonly reported symptoms [17]. The reported interval between the initial diagnosis of cervical cancer and presentation of the brain metastasis is variable in different cases, ranging from the time of first diagnosis of the primary tumor to 8 years, with an overall mean of 3 months [18]. In our case, brain metastases were diagnosed concomitantly with cervical cancer diagnosis.

Ours is perhaps the second reported case of cavernous sinus metastasis from primary uterine cervical cancer in the English literature with the other documented case reported by Tsuda and colleagues [19]. Similar to our case, abducens nerve palsy was a presentation with ipsilateral Horner syndrome as an additional presentation with concomitant diagnosis of uterine cervical cancer in this case. Like other case reports with brain metastases from cervical cancer, our case also had abrupt onset of symptoms and wide dissemination with lung, retroperitoneal and skin involvement as mentioned in some studies [20, 21].

Favorable prognostic indicators in patients with brain metastasis from cervical cancer include young age (< 65 years), good performance status, absence of or limited extracranial disease and fewer number of metastases [22]. Unfortunately our case had none of the favorable features with the exception of her age.

There is no standard treatment for brain metastasis from cervical cancer [23]. Surgery is suitable for patients with single metastases in the absence of extracranial disease and when there is mass effect. Whole brain radiation therapy (WBRT) should be considered for these patients and it helps to improve local control [4-10]. Palliative treatment with steroids is not beneficial because the patients usually do not survive more than 1 month and most die of neurologic problems. WBRT alone is the treatment of choice for multiple metastases in brain than combination chemotherapy [23]. It is believed that intravenous chemotherapy does not cross the blood-brain barrier. The aim of radiotherapy is palliation in such cases, and the radiation doses range from 8 Gy in single fraction to 40 Gy/15 fractions [8]. Our intention in treating this patient was also palliation, and thus a dose of 30 Gy/10 fractions/2 weeks plus a boost dose of 5 Gy/2 fractions to the cavernous sinus lesion was prescribed with steroids, but the patient could not complete the treatment as prescribed because of poor performance status and rapid decline in her symptoms.

Stereotactic radiosurgery such as cyberknife is as effective as conventional surgery and can be used in patients with surgically inaccessible lesions. Unfortunately, most brain metastases are multifocal and accompanied by other organ metastasis [20, 21, 24], as in our case. Brain metastases from cervical cancer carry a poor prognosis. Most studies have reported a median survival of only a few months, but there are a few anecdotal reports of long-term, disease-free survival in these patients [8, 25-28]. Our patient survived only a month after WBRT.

In conclusion, though brain metastases from uterine cervical cancer are uncommon, it should always be considered in the scenario of advanced uterine cervical cancer. Cavernous sinus metastasis from uterine cervical cancer is even rarer, the present case being the second documented case of this clinical phenomenon in the English literature. Therefore, if signs and symptoms of cavernous sinus pathology are present in the setting of advanced uterine cervical cancer, the possibility of metastasis to this site from the primary cervical cancer should be raised and further investigated upon.
